# Diclofenac-induced biochemical and histopathological changes in white leghorn birds (*Gallus domesticus*)

**DOI:** 10.4103/0253-7613.58515

**Published:** 2009-10

**Authors:** Teenu Jain, K.M. Koley, V.P. Vadlamudi, R.C. Ghosh, S. Roy, Sandhya Tiwari, Upasana Sahu

**Affiliations:** Department of Veterinary Pharmacology and Toxicology, College of Veterinary science & AH, Anjora (IGAU), Durg - 491 001, Chhattisgarh, India

**Keywords:** Diclofenac, visceral gout, nephrotoxicity, White Leghorn birds

## Abstract

**Objective:**

Objective: To evaluate diclofenac-induced biochemical and histopathological changes in White Leghorn birds.

**Materials and Methods:**

Six-week-old birds were equally divided into three groups of six birds each. Group I served as control and received vehicle orally. The birds of Group II and III were orally administered with a single low (2 mg/kg) and high dose (20 mg/kg) of diclofenac sodium, respectively, and were observed for 7 days. The acute toxicity was assessed by observing the clinical signs and symptoms, mortality, alterations in blood biochemistry, and necropsy findings.

**Results:**

The birds of Group II showed only mild symptoms of diarrhea. In Group III, 50% of birds died in between 24 and 36 h post-treatment showing the symptoms of segregatory behavior, lethargy, terminal anorexia, and severe bloody diarrhea. The birds of Group II and the surviving birds of Group III showed a significantly (P<0.05) increased plasma uric acid, creatinine and plasma glutamic pyruvic transaminase (PGPT), and decreased total protein and albumin at 12 and 24 h post-treatment which returned to the normal levels at 36 h post-treatment. The dead birds of the high-dose group also showed similar pattern of biochemical changes at 12 and 24 h post-treatment and revealed extensive visceral gout with characteristic histopathological lesions in liver, kidney, heart, spleen, and intestine on post-mortem.

**Conclusion:**

The results indicate that diclofenac sodium has hepatotoxic, nephrotoxic, and visceral gout inducing potentials in White Leghorn birds, especially at higher dose.

## Introduction

NSAIDs are characterized by the ability to inhibit cyclo-oxygenase enzymes, which are involved in the formation of prostaglandins. However, there are marked differences between drugs in their selective inhibition of the two subtypes of cyclo-oxygenase, COX-1 and COX-2, the latter being involved with the modulation of inflammation-mediated responses and pain, while the former modulates blood flow to the kidneys. The ability of NSAIDs to inhibit both these subtypes has been implicated as a cause of the side effects occasionally associated with the use of some NSAIDs. Toxic effects on the kidneys of vultures have been observed with number of NSAIDs.[1] The widespread use of diclofenac sodium in veterinary medicine has been linked to near extinction of vultures in the Indian subcontinent, and as such the drug has been withdrawn from veterinary use in the year 2006. Vulture deaths were reported to be due to severe renal damage causing visceral gout following scavenging on dead livestock treated shortly before death.[2] The aetiopathology of diclofenac-induced visceral gout in vultures is not conclusively understood. Though there are no published reports of using diclofenac as such in poultry birds, a wide variety of indications (trauma, coccidiosis, heat stress, and pain related to beak trimming)[3–6] exist for which treatment with other NSAIDs could be beneficial in bird medicine. The present study was planned with the objective to evaluate the acute oral toxicity of diclofenac sodium in White Leghorn birds and to assess whether diclofenac is also similarly toxic to these birds as reported in vultures.

## Materials and Methods

### Experimental animals

Eighteen adult healthy birds were divided into three groups of six each. They were fed standard broiler feed and water *ad libitum*. All the birds were fasted overnight and weighed individually just prior to administration of the test drug.

### Drug used

Diclofenac sodium (Voveran® tablets containing 50 mg diclofenac sodium IP of Novartis India Limited, Pune) was used. The tablets were powdered and orally administered as aqueous suspension in a 1% solution of sodium carboxymethyl cellulose. Control birds received only vehicle. Groups II and III birds received a single oral dose of diclofenac sodium, 2 mg/ kg (approximate mammalian therapeutic dose) and 20 mg/kg (high dose), respectively.

### Samples

Whole blood (2 ml) was collected from the jugular/wing vein in heparinized vials at 0, 12, 24, and 36 h post-treatment. Plasma was separated immediately by centrifugation and stored at −20°C until analyzed.

After drug administration, the birds were observed for onset, nature, and severity of clinical symptoms and mortality, if any, up to seventh day post-administration. Blood samples were also analyzed for estimation of total protein, albumin, uric acid, creatinine, and plasma glutamic pyruvate transaminase (PGPT) in plasma by Bayer's reagent kits using ROBONIK's Semi-autoanalyzer at pre-treatment and 12, 24, and 36 h post-treatment.

All the birds, which died due to toxicity of diclofenac in Group III, were autopsied and the surviving birds of Groups II and III were sacrificed and subjected to pathological investigations.

### Statistical analysis

The statistical analysis was performed using two-way ANOVA followed by Duncan's multiple range test. The significance in difference was accepted at *P* < 0.05.[7]

## Results

### General observations

Birds of Group I did not show any signs and symptoms of toxicity. Birds of Group II showed only mild diarrhea. In Group III, 50% of birds succumbed to toxicity between 24 and 36 h post-treatment and showed dullness, segregatory behavior, blood tinged diarrhea, and anorexia before death. Survived birds of this group had only blood-tinged diarrhea.

### Biochemical changes

Effect on plasma uric acid, creatinine, and glutamic pryruvic transaminase

Birds of Groups II and III (survived) showed significantly elevated uric acid, creatinine, and PGPT levels at 12 and 24 h of post-treatment as compared to control which returned to normal levels at 36 h post-treatment. Group III birds that succumbed to toxicity showed elevated parameters at 12 and 24 h [Tables 1 and 2].

**Table 1:** Effect of single oral dose of diclofenac sodium on plasma uric acid levels and creatinine in white leghorn birds

*Dose of diclofenac sodium*	*Mean plasma uric acid (mg/dl)*				*Mean plasma creatinine (mg/dl)*			
	
	*Pre-treatment*	*Post-treatment*			*Pre-treatment*	*Post-treatment*		
	
		*12 h*	*24 h*	*36 h*		*12 h*	*24 h*	*36 h*
Control	6.18^a,d^ ± 0.62 (6)	6.66^a^ ± 0.58 (6)	6.68^a^ ± 0.45 (6)	6.58^a,f^ ± 0.56 (6)	0.60^g,m^ ± 0.04 (6)	0.60^g^ ± 0.03 (6)	0.58^g^ ± 0.03 (6)	0.61^g, n^ ± 0.02 (6)
2 mg/kg	6.18^b,d^ ± 0.62 (6)	19.81 ± 0.41 (6)	11.03^e^ ± 0.99 (6)	7.43^b,f^ ± 0.68 (6)	0.60^h,m^ ± .04 (6)	0.90 ± .03 (6)	0.73^i^ ± 0.01(6)	0.64^h,i,n^ ± 0.02 (6)
20 mg/kg								
Live birds	5.06^c,d^ ± 0.18 (3)	24.50 ± 0.73 (3)	12.60^e^ ± 0.49 (3)	0.56^d,f^ ± 0.08 (3)	0.56^j,m^ ± 0.08 (3)	1.08^k^ ± 0.04 (3)	0.99^k^ ± 0.00 (3)	0.69^j,n^ ± 0.01 (3)
Dead birds	7.3^d^ ± 0.55 (3)	26.43 ± 0.93 (3)	39.93 ± 1.79 (3)	0.63^f^ ± 0.03 (3)	0.63^m^ ± 0.03 (3)	1.35 ± 0.12 (3)	1.56 ± 0.03 (3)	Died

*P* < 0.05. Mean values with similar superscripts within the rows and columns are statistically similar. Figures within parentheses refer to number of observations

**Table 2:** Effect of single oral dose of diclofenac sodium on plasma glutamic pyruvic transaminase in white leghorn birds

*Dose of diclofenac sodium*	*Mean plasma glutamic pyruvic transaminase (U/l) ± SE*			
	
	*Pre-treatment (0 h)*	*Post-treatment (h)*		
		
		*12*	*24*	*36*
Control	8.01^a,d^ ± 0.84 (6)	8.7^a^ ± 0.79 (6)	9.1^a,e^ ± 0.72 (6)	9.3^a,g^ ± 0.58 (6)
2 mg/kg	8.01^b,d^ ± 0.84 (6)	18.18 ± 1.56 (6)	11.93^e,f^ ± 0.71 (6)	7.02^b,g^ ± 0.41 (6)
20 mg/kg				
Live birds	8.27^c,d^ ± 1.07 (3)	21.83 ± 1.43 (3)	14.33^f^ ± 0.51 (3)	7.5^c,g^ ± 0.39 (3)
Dead birds	7.76^d^ ± 0.75 (3)	28.33 ± 0.84 (3)	37.83 ± 0.96 (3)	7.5^c,g^ ± 0.39 (3)

*P* < 0.05, Mean values with similar superscripts within the rows and columns are statistically similar.

Effect on plasma total protein and albumin

Birds of Groups II and III (those survived) showed significantly lowered total protein and plasma albumin levels at 12 and 24 h of post-treatment as compared to control that returned to normal levels at 36 h post-treatment. Group III birds which succumbed to toxicity, showed lowered parameters at 12 and 24 h [Table 3].

**Table 3:** Effect of single oral dose of diclofenac sodium on total plasma protein and albumin in white leghorn birds

*Dose of diclofenac sodium*	*Mean plasma uric acid (mg/dl)*				*Mean plasma creatinine (mg/dl)*			
	
	*Pre-treatment*	*Post-treatment*			*Pre-treatment*	*Post-treatment*		
			
		*12 h*	*24 h*	*36 h*		*12 h*	*24 h*	*36 h*
Control	4.88^a,c^ ± 0.15(6)	4.55^a^ ± 0.14(6)	4.75^a^ ± 0.20(6)	4.88^a,f^ ± 0.15(6)	2.23^g,l^ ± 0.01(6)	2.40^g^ ± 0.13(6)	2.70^g,n^ ± 0.09(6)	2.34^g,o^ ± 0.15(6)
2 mg/kg	4.88^b,c^ ± 0.21(6)	1.93^d^ ± 0.08 (6)	2.68^e^ ± 0.20(6)	4.16^b,f^ ± 0.20(6)	2.23^h,l^ ± 0.01(6)	1.64^i^ ± 0.08(6)	1.97^i,n^ ± 0.02(6)	2.23^h,o^ ± 0.01(6)
20 mg/kg								
Live birds	4.83^c^ ± 0.33(3)	1.66^d^ ± 0.02(3)	3.11^e^ ± 0.06(3)	3.97^f^ ± 0.25(3)	2.07^j,l^ ± 0.16(3)	1.29^j,m^ ± 0.08(3)	2.33^j,n^ ± 0.17(3)	2.45^j,o^ ± 0.08(3)
Dead birds	4.93^c^ ± 0.06 (3)	1.66^d^ ± 0.08(3)	1.07 ± 0.03 (3)	3.97^f^ ± 0.25(3)	2.40^l^ ± 0.05 (3)	1.01^k,m^ ± 0.01 (3)	0.91^k^ ± 0.03 (3)	Died

*P* < 0.05, Mean values with similar superscripts within the rows and columns are statistically similar. Figures within parentheses refer to number of observation within parentheses refer to number of observations.

### Pathological changes

Gross pathology

**Figure 1:**
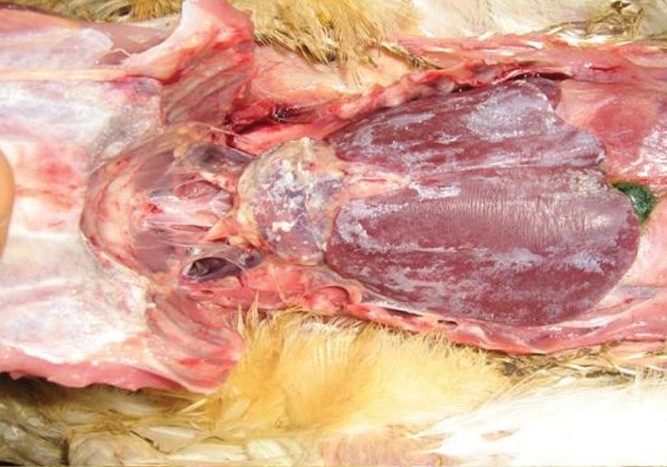
Viscera of dead bird of group III showing chalky deposition of uric acid

Groups I and II birds did not show any gross pathological lesions. The dead birds of Group III on post-mortem showed extensive visceral gout characterized by chalky white deposition on the serosal surface [Figure 1]. This deposit on the serosal surface was confirmed to be uric acid by the Murexide test.[8] Survived birds of Group III showed only sporadic congestion in the intestine.

Histopathology

The liver, kidney, heart, spleen, and intestine sections of Group I birds did not show any histopathological lesions.

Liver

**Figure 2:**
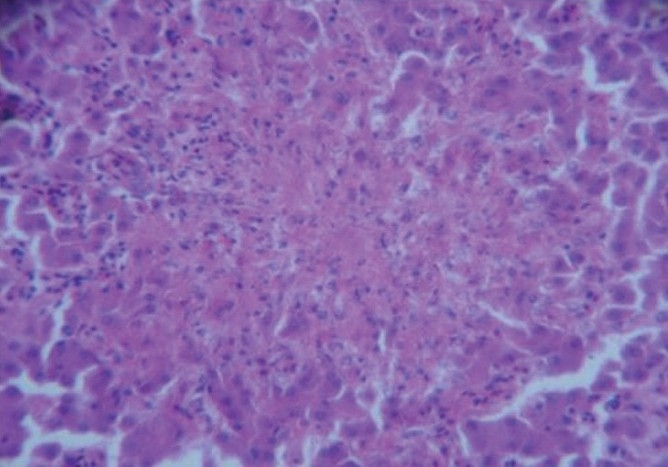
Liver section of dead bird of group III showing severe necrosis with mononuclear cell infiltration (H & E, ×400)

The liver section of Group II birds showed mild hydropic degeneration. The section of survived bird of Group III exhibited periportal fibrosis and focal aggregation of lymphocytes. Section of birds, which succumbed to toxicity, showed severe necrosis with infiltration of mononuclear cells [Figure 2].

Kidney

**Figure 3:**
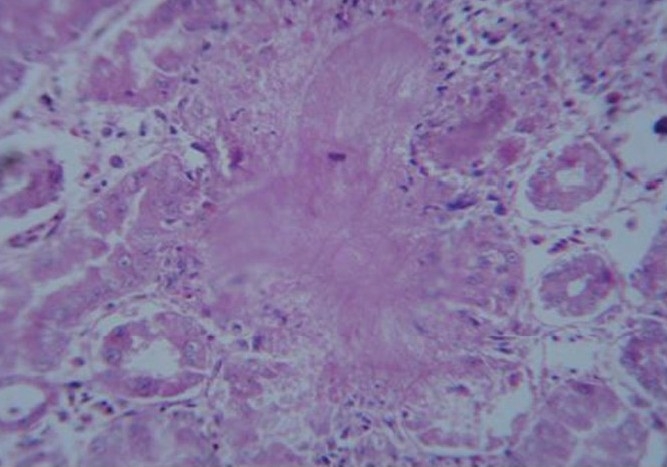
Kidney section of dead bird of group III showing radiating uric acid crystals (H & E, ×400)

The kidney section of birds of Group II showed mild degenerative changes in the renal tubules. The section of survived birds of Group III showed mild degenerative and necrotic changes in the renal tubules along with infiltration of mononuclear cells in the intertubular areas. The section of birds of Group III which succumbed to toxicity showed tubular degeneration and varying sized foci of urate deposition (tophi) either in the form of amorphous material or in the radiating crystalline pattern mixed with necrotic debri due to degeneration of surrounding cells [Figure 3]

Heart, Spleen, Intestine

**Figure 4:**
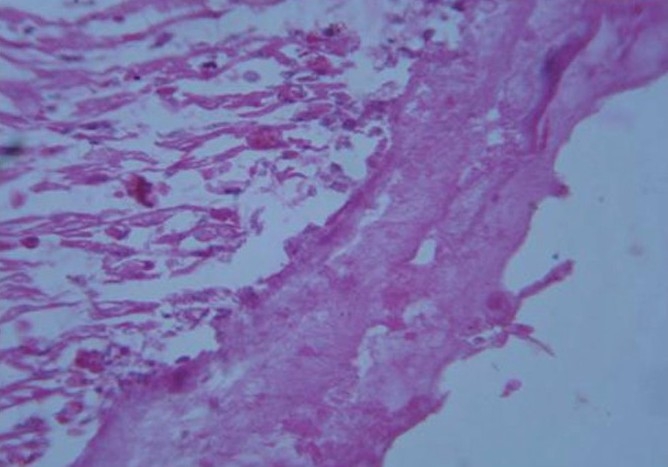
Heart section of dead bird of group III showing thickening of epicardium (H & E, ×400)

**Figure 5:**
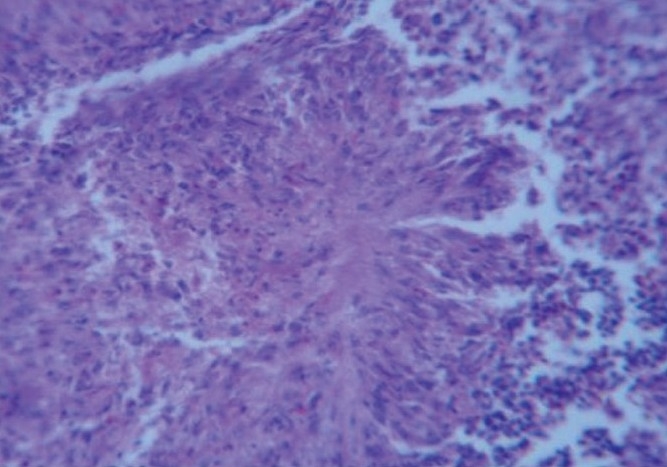
Spleen section of dead bird of group III showing radiating necrotic mass indicating area deposited with urate crystals (H & E, ×400)

The heart, spleen, and intestine sections of birds of Group II and survived birds of Group III did not show any lesions. The heart section of birds of Group III that succumbed to toxicity showed necrosis of muscle fiber, edema, and thickening of epicardium which might be due to the presence of acellular eosinophilic material [Figure 4]. The spleen section from the same group showed depletion of lymphocytes from the white pulp of spleen and radiating necrotic mass indicating area deposited with urate crystals [Figure 5] and only congestion was seen in the mucosal layer of intestine.

## Discussion

The acute toxicity of diclofenac sodium was studied by many workers in avian species. 40% mortality was observed in Vanraja and PB1 birds within 12 days following inoculation of diclofenac sodium in the dose of 5 mg/kg.[9] Diclofenac sodium, 0.8 mg/ kg was highly toxic to *Gyps fulvus* and *G. africanus* species of vultures and the oral LD_50_ of diclofenac in *G. bengalensis* was found to be 0.1-0.2 mg/kg.[10] In the present study, a single dose of 20 mg/kg, p.o., caused 50% mortality in White Leghorn birds. The results indicate that diclofenac is also toxic to these birds but they are less sensitive as compared to reports in vultures.

Clinical gout in chicken was reported when serum uric acid level exceeded 25.5 mg/dl as compared to 4.2-6.4 mg/dl in the control birds.[11] the increased level of uric acid was found as high as 140 and 160 mg/dl, at 12 and 24 h after administration of diclofenac, 0.8 mg/kg orally in *G. Africanus* vultures.[10]

The increased uric acid level observed in this study, following diclofenac administration might be due to renal failure which leads to hyperuricemia and gout because kidneys plays an important role in elimination of uric acid and creatinine in birds. Blood flow to the avian kidney is different from that of mammals. In mammalian kidney, PGE_2_ and PGI_2_ function as renal vasodilators and regulate renal blood flow supplied primarily through the afferent arteriole.[12] Diclofenac is a powerful inhibitor of cyclo-oxygenase and prostaglandin synthetase both of which are involved in PGE_2_ production. Diclofenac blocks PGE_2_ synthesis through inhibition of COX.[13] However, in avian kidney, the renal portal system via the afferent renal portal vein is the primary nutrient blood source for the renal cortex and does not supply the renal medulla and medullary cone.[14] The primary blood supply to avian glomeruli and DCT is central artery. Blood is supplied to the PCT via renal portal system. The effect of diclofenac on this system may be responsible for higher sensitivity of birds to its renal toxicity than mammals.

The mechanism of diclofenac-induced renal failure in Oriental White Backed Vultures (OWBVs) has been proposed to be through the inhibition of the modulating effect of prostaglandin on angiotensin II-mediated adrenergic stimulation. Blood flow to the renal cortex is under multiple levels of adrenergic control. The smooth muscle sphincter of renal portal valve are highly innervated by adrenergic and choloinergic nerves. Renal portal valve opens in response to adrenergic stimulation, redirecting portal blood to the caudal venacava bypassing the kidney. Therefore, interference in prostaglandin modulation on renal portal valves by diclofenac might be resulting in indiscriminate action of the valves redirecting the primary nutrient blood supply away from the renal cortex leading to ischemic necrosis of PCT.[15]

Necrosis of PCT would compromise uric acid excretion, leading to rapid elevation of uric acid concentration in blood. Once the saturation point is reached in the blood, uric acid would rapidly precipitate as crystals on the organ surface and within organ parenchyma resulting in death. Diclofenac affects kidney of various genera of birds differentially. The possibility of differential physiologic response to diclofenac may also play a role in differential toxicity. There is species variation in the anatomy of the renal portal system and species anatomy of these structures is still unknown.[16]

The roles of PGE_2,_ PGI_2_ and COX-1 in smooth muscle control of renal portal valve and its modification by diclofenac in White Leghorn birds merits further studies.

Creatinine after being filtered by glomerulus is excreted in urine. Since it is not excreted or absorbed by renal tubules to any degree, it can be used as a rough index of GFR.[17] Increased level of plasma creatinine observed in this study might be related to blockade of renal vasodilatation due to nonselective inhibition of the cyclo-oxygenase by diclofenac sodium.

In this study, the elevated plasma GPT activity, decrease in plasma protein, albumin, and histopathological changes in liver section are suggestive of hepatic damage.[18,19] Diclofenac causes a rare but potentially fatal hepatotoxicity that may be associated with the formation of reactive metabolites (benzoquinones imines) via a hepatic cytochrome P450 catalyzed oxidation which contribute to diclofenac-mediated hepatic injury.[20,21] The histopathological changes observed in the present study on various organs are similar to the already existing reports.[2,11,15,22–26]

## Conclusion

The biochemical and pathological alterations observed in this study suggest that diclofenac sodium at a higher dose has hepatotoxic, nephrotoxic, and visceral gout inducing potential in White Leghorn birds but as compared to reports in vultures they seem to be less sensitive.
